# Is Exercise an Efficacious Treatment for Depression? A Comment upon Recent Negative Findings

**DOI:** 10.3389/fpsyt.2013.00020

**Published:** 2013-04-02

**Authors:** Felipe Barreto Schuch, Marcelo Pio de Almeida Fleck

**Affiliations:** ^1^Post-Graduate Program in Medical Science: Psychiatry, Hospital de Clínicas de Porto Alegre, Universidade Federal do Rio Grande do SulPorto Alegre, Brazil

Exercise is receiving substantial and increasing attention as a potential treatment for depression. Despite the many positive meta-analytical findings and recommendations of some guidelines to incorporate exercise as a treatment for depression (National Institute for Health and Clinical Excellence, 2009), most clinical trials have significant methodological flaws that limit the generalizability of their findings (Daley, 2008; Rethorst et al., 2009). Furthermore, recent meta-analyses show that, when only robust clinical trials are included, the effects of exercise are “moderate at best” or statistically insignificant (Rimer et al., 2012).

Similarly, four recent, robust, randomized controlled trials (RCTs) have failed to find any antidepressant effects of exercise: the DEMO (Krogh et al., 2009), DEMO II (Krogh et al., 2012), TReatment with Exercise Augmentation for Depression (TREAD) (Trivedi et al., 2011), and TREAD-UK (Chalder et al., 2012). The DEMO trial (Krogh et al., 2009) compared aerobic exercise, anaerobic exercise, and relaxation (control) groups; after 4 months of intervention, no differences in depressive symptoms, as assessed by the Hamilton scale for depression (HAM-D17) (Hamilton, 1967), were found between the groups. Subsequently, the DEMO II trial (Krogh et al., 2012) compared aerobic exercise and stretching (control) groups; after 3 months, the authors found reductions in HAM-D17 scores in both groups but failed to find differences between the groups. In both studies, the authors concluded that exercise was as effective as placebo and had no biological antidepressant effects.

Corroborating the DEMO and DEMO II findings, the TREAD (Trivedi et al., 2011) found a non-significant trend (*p* < 0.06) toward a remission rate of 16 kcal/kg/week for aerobic exercise versus 4 kcal/kg/week for aerobic exercise (control) after 3 months of intervention. Lastly, the TREAting Depression with physical activity (TREAD-UK) (Chalder et al., 2012) evaluated the cost-effectiveness of a strategy for promoting and increasing the physical activity levels of depressed patients in primary care. After 8 months, physical activity was shown to be an ineffective and more costly strategy than conventional primary care assistance and resulted in a non-cost-effective strategy according to willingness-to-pay thresholds.

Considering these recent results, the answer to the question “is exercise an efficacious treatment for depression?” appears to be “No.” However, before this question is answered, some issues must be highlighted.

Similar to the findings regarding exercise, several meta-analyses have shown that the benefits of antidepressants are “minimal or non-existent,” as they are as effective as placebo. Other meta-analyses have shown that antidepressants are effective only in severe, but not in moderate or mild depression (Kirsch et al., 2008; Fournier et al., 2010; Khan et al., 2012). On the other hand, clinical practice and some qualitative studies have revealed that both exercise and antidepressants are effective in the opinion of patients (Searle et al., 2011) and mental health professionals (Martinsen, 1994; Kirsch, 2008); these findings reveal a discrepancy between the views of health professionals and patients and the clinical findings.

There are some possible explanations for this discrepancy: (1) the heterogeneity of “depression” as a construct; (2) the psychometric pitfalls of depression assessment scales; and (3) the possible interference of unspecific factors that could mask the biological effects of exercise.

Parker (2005) argued that the “major depression” concept classifies a heterogeneous group of patients who may have different, and sometimes opposite, clinical features and symptoms (e.g., insomnia or hypersomnia, weight loss or weight gain, and psychomotor retardation or psychomotor agitation) into on diagnosis. In the words of Parker, the major depression construct “circumscribes a range of heterogeneous conditions, homogenizes them, and has come to be viewed as an entity.” This quote exemplifies the diagnoses of individuals with “clinical dyspnea” because such diagnoses are not informative because the individual may have asthma, pneumonia, or a pulmonary embolus, and each condition requires a different treatment. Similarly, major depression may need a more finely focused diagnosis that requires greater explanatory power. The proposal of Parker is a new categorical-dimensional model that classifies three more finely focused diagnostic subgroups: psychotic depression, melancholic depression, and non-melancholic depression; these subgroups possibly have different biological backgrounds and may be responsive to different treatments. For more detailed information, see Parker's works concerning a new categorical-dimensional construct of depression.

Another possible explanation for this discrepancy may be the low “effectivity” of the HAM-D17, which is the most used instrument in RCTs and was used in three of the recent studies of exercise (DEMO, DEMO II, and TREAD). Some studies have shown that this scale has several flaws, as assessed by classical (Bagby et al., 2004; Fleck et al., 2004) and modern psychometric techniques, including Item response theory and Rasch analyses (Bagby et al., 2004). Based upon these studies, the HAM-D17 lacks uni-dimensionality; furthermore, some items appear to be insufficiently discriminative of different levels of depression, particularly within the mild and moderate levels, although the HAM-D17s work for severe levels of depression (Santor and Coyne, 2001). According Salum et al. (2011), the HAM-D17 is like an “industrial thermometer being used to measure a temperature of a baby.” Interestingly, antidepressants have higher efficacy in severe, compared to low or moderate, depression (Kirsch et al., 2008; Fournier et al., 2010). Furthermore, some preliminary results show that exercise is also effective as a complementary treatment of severely depressed inpatients (Knubben et al., 2007; Schuch et al., 2011).

Finally, exercise has many factors that “compose” the exercise session but are not exercise *per se*. For example, when a patient exercises in a group setting, or even in the company of health professionals, the resultant social support and attention may have an important role, especially in light depression. A similar effect occurs in structured psychotherapies studies, in which non-structured interventions (placebos) efficacies similar to structured interventions (Jakobsen et al., 2011).

Before a final judgment is made, clinical trials using exercise as a treatment for depression must be carefully analyzed. Furthermore, meta-analyses show that exercise has similar efficacy as other standardized treatments for depression including some antidepressants and some psychotherapies. Thus, exercise may not be more efficacious than conventional treatments; however, it is not less efficacious.

On the other hand, exercise has other issues that deserve more attention: the initial acceptance and compliance/adherence. Two of the major difficulties of the use of exercise as a treatment for depressed patients are the initial acceptance of and compliance/adherence to exercise regimens. For example, two weaknesses of the DEMO II trial are the limited number of patients included and the low compliance with the exercise sessions.

In the DEMO trial, of 390 possible patients recruited, 100 refused to participate. In the DEMO II trial, the patients’ inclusion had to stop due to “lower referral than anticipated.” Second, the authors’ initial sample size tests showed that 85 subjects in each group were necessary, but after the recruitment phase and an additional 12 months of recruitment, they had just 56 subjects in the aerobic exercise group and 59 in the stretching group. Lastly, in our study (unpublished data), 46 of 96 severely depressed inpatients refused to participate in the study, and, of these 46, 40 stated they had low interest in exercise.

Moreover, the in DEMO II trial, patients attended a mean 13.5 and 12.5 of 36 sessions of aerobic exercise and stretching, respectively. In an attempt to increase adherence/compliance, Trivedi et al. (2006b) suggested that a more flexible program based upon energetic expenditure, without a fixed intensity (e.g., 70% maximum heart rate), may be more suitable and increase compliance. For example, in the DOSE study (Trivedi et al., 2006a), patients in the exercise groups completed 72% of the sessions. In another recent study, Callaghan et al. (2011) showed that women that exercised “as recommended by national guidelines” attended 6 of 12 sessions (50%), while the group that had exercised at the “preferred intensity” attended 8 of 12 sessions (66%), resulting in a mean increase of 2/12 sessions. In the TREAD (Trivedi et al., 2011) study, the low and high dose groups had mean adherences of 99.4 and 63.8%, respectively. These three studies show that more flexible strategies may be an interesting alternative that may increase adherence and compliance compared to conventional strategies (e.g., 30 min at 70% of maximal heart rate).

Two interesting papers propose some practical suggestions to initiate and sustain exercise and physical activity in depressed patients. One is a paper from Seime and Vickers (2006) that suggests some practical strategies including the following: promote discussions related to what activities “would benefit your patient most based upon his/her symptoms” in terms of pre-preferences and personal barriers that prevent the patient from beginning or maintaining exercise, and share with patients an easily accessible brief overview of recommendations for physical activity that could include the guideline recommendations for public health and the dose-response relationship demonstrated by Dunn et al. (2005). Moreover, Blumenthal et al. (2012) suggested that exercise professionals should become more familiar with the principles of motivational interviewing, a well-researched approach to promoting behavioral change, and the “seven tips” for patients.

In summary, recent negative findings concerning the use of exercise as a treatment for depressed patients must be interpreted with caution. Exercise, along with other treatments for depression, has some issues because the instrument used to assess depression and the diagnoses of depression may lead to misunderstandings. Thus, the DSM-V will most likely open new perspectives concerning diagnoses of depression, and subsequently, the instruments used to assess depression. Although the initial adherence and compliance with exercise programs are a great challenge, the use of cognitive and motivational approaches and more flexible and comprehensive strategies of exercise may be useful in increasing and maintaining a the exercise of depressed patients.

## References

[B1] Bagby R. M. Ryder A. G. Schuller D. R. Marshall M. B. (2004). The Hamilton Depression Rating Scale: has the gold standard become a lead weight? Am. J. Psychiatry 161, 2163–2177 10.1176/appi.ajp.161.12.2163 15569884

[B2] Blumenthal J. A. Smith P. J. Hoffman B. M. (2012). Opinion and evidence: is exercise a viable treatment for depression? ACSMs Health Fit. J. 16, 14–21 10.1249/01.FIT.0000416000.09526.eb PMC3674785 23750100

[B3] Callaghan P. Khalil E. Morres I. Carter T. (2011). Pragmatic randomised controlled trial of preferred intensity exercise in women living with depression. BMC Public Health 11:465 10.1186/1471-2458-11-465 21663696 PMC3128029

[B4] Chalder M. Wiles N. J. Campbell J. Hollinghurst S. P. Searle A. Haase A. M. (2012). A pragmatic randomised controlled trial to evaluate the cost-effectiveness of a physical activity intervention as a treatment for depression: the treating depression with physical activity (TREAD) trial. Health Technol. Assess. 16, 1–164 22398106 10.3310/hta16100

[B5] Daley A. (2008). Exercise and depression: a review of reviews. J. Clin. Psychol. Med. Settings 15, 140–147 10.1007/s10880-008-9105-z 19104978

[B6] Dunn A. L. Trivedi M. H. Kampert J. B. Clark C. G. Chambliss H. O. (2005). Exercise treatment for depression: efficacy and dose response. Am. J. Prev. Med. 28, 1–8 10.1016/j.amepre.2004.09.003 15626549

[B7] Fleck M. P. Chaves M. L. Poirier-Littre M. F. Bourdel M. C. Loo H. Guelfi J. D. (2004). Depression in France and Brazil: factorial structure of the 17-item Hamilton Depression Scale in inpatients. J. Nerv. Ment. Dis. 192, 103–110 10.1097/01.nmd.0000110281.35970.33 14770054

[B8] Fournier J. C. Derubeis R. J. Hollon S. D. Dimidjian S. Amsterdam J. D. Shelton R. C. (2010). Antidepressant drug effects and depression severity: a patient-level meta-analysis. JAMA 303, 47–53 10.1001/jama.2009.1943 20051569 PMC3712503

[B9] Hamilton M. (1967). Development of a rating scale for primary depressive illness. Br. J. Soc. Clin. Psychol. 6, 278–296 10.1111/j.2044-8260.1967.tb00530.x 6080235

[B10] Jakobsen J. C. Hansen J. L. Storebo O. J. Simonsen E. Gluud C. (2011). The effects of cognitive therapy versus ‘no intervention’ for major depressive disorder. PLoS ONE 6:e28299 10.1371/journal.pone.0028299 22174786 PMC3235113

[B11] Khan A. Faucett J. Lichtenberg P. Kirsch I. Brown W. A. (2012). A systematic review of comparative efficacy of treatments and controls for depression. PLoS ONE 7:e41778 10.1371/journal.pone.0041778 22860015 PMC3408478

[B12] Kirsch I. (2008). Antidepressant drugs ‘work,’ but they are not clinically effective. Br. J. Hosp. Med. (Lond.) 69, 359 18646426

[B13] Kirsch I. Deacon B. J. Huedo-Medina T. B. Scoboria A. Moore T. J. Johnson B. T. (2008). Initial severity and antidepressant benefits: a meta-analysis of data submitted to the Food and Drug Administration. PLoS Med. 5:e45 10.1371/journal.pmed.0050045 18303940 PMC2253608

[B14] Knubben K. Reischies F. M. Adli M. Schlattmann P. Bauer M. Dimeo F. (2007). A randomised, controlled study on the effects of a short-term endurance training programme in patients with major depression. Br. J. Sports Med. 41, 29–33 10.1136/bjsm.2006.030130 17062659 PMC2465130

[B15] Krogh J. Saltin B. Gluud C. Nordentoft M. (2009). The DEMO trial: a randomized, parallel-group, observer-blinded clinical trial of strength versus aerobic versus relaxation training for patients with mild to moderate depression. J. Clin. Psychiatry 70, 790–800 10.4088/JCP.08m04241 19573478

[B16] Krogh J. Videbech P. Thomsen C. Gluud C. Nordentoft M. (2012). DEMO-II trial. Aerobic exercise versus stretching exercise in patients with major depression-a randomised clinical trial. PLoS ONE 7:e48316 10.1371/journal.pone.0048316 23118981 PMC3485141

[B17] Martinsen E. W. (1994). Physical activity and depression: clinical experience. Acta Psychiatr. Scand. Suppl. 377, 23–27 10.1111/j.1600-0447.1994.tb05797.x 8053362

[B18] National Institute for Health Clinical Excellence. (2009). Depression: the treatment and management of depression in adults (update). Available at: http://www.nice.org.uk/guidance/CG90

[B19] Parker G. (2005). Beyond major depression. Psychol. Med. 35, 467–474 10.1017/S0033291704004210 15856717

[B20] Rethorst C. D. Wipfli B. M. Landers D. M. (2009). The antidepressive effects of exercise: a meta-analysis of randomized trials. Sports Med. 39, 491–511 10.2165/00007256-200939060-00004 19453207

[B21] Rimer J. Dwan K. Lawlor D. A. Greig C. A. McMurdo M. Morley W. (2012). Exercise for depression. Cochrane Database Syst. Rev. 7, CD004366 22786489 10.1002/14651858.CD004366.pub5

[B22] Salum G. A. Manfro G. G. Fleck M. P. (2011). What is not “effective” in mild to moderate depression: antidepressants or the Hamilton Rating Scale for depression? CNS Spectr. [Epub ahead of print]. 24725372 10.1017/S1092852912000259

[B23] Santor D. A. Coyne J. C. (2001). Examining symptom expression as a function of symptom severity: item performance on the Hamilton Rating Scale for Depression. Psychol. Assess. 13, 127–139 10.1037/1040-3590.13.1.127 11281034

[B24] Schuch F. B. Vasconcelos-Moreno M. P. Borowsky C. Fleck M. P. (2011). Exercise and severe depression: preliminary results of an add-on study. J. Affect. Disord. 133, 615–618 10.1016/j.jad.2011.04.030 21616540

[B25] Searle A. Calnan M. Lewis G. Campbell J. Taylor A. Turner K. (2011). Patients’ views of physical activity as treatment for depression: a qualitative study. Br. J. Gen. Pract. 61, 149–156 10.3399/bjgp11X567054 21439172 PMC3063043

[B26] Seime R. J. Vickers K. S. (2006). The challenges of treating depression with exercise: from evidence to practice. Clin. Psychol. 13, 194–197

[B27] Trivedi M. H. Greer T. L. Church T. S. Carmody T. J. Grannemann B. D. Galper D. I. (2011). Exercise as an augmentation treatment for nonremitted major depressive disorder: a randomized, parallel dose comparison. J. Clin. Psychiatry 72, 677–684 10.4088/JCP.11m06837 21658349 PMC9900872

[B28] Trivedi M. H. Greer T. L. Grannemann B. D. Chambliss H. O. Jordan A. N. (2006a). Exercise as an augmentation strategy for treatment of major depression. J. Psychiatr. Pract. 12, 205–213 10.1097/00131746-200607000-00002 16883145

[B29] Trivedi M. H. Greer T. L. Grannemann B. D. Church T. S. Galper D. I. Sunderajan P. (2006b). TREAD: treatment with exercise augmentation for depression: study rationale and design. Clin. Trials 3, 291–305 10.1191/1740774506cn151oa 16895046

